# A Comparison of Non-verbal Maternal Care of Male and Female Infants in India and the United Kingdom: The Parent-Infant Caregiving Touch Scale in Two Cultures

**DOI:** 10.3389/fpsyg.2022.852618

**Published:** 2022-03-23

**Authors:** John Hodsoll, Andrew Pickles, Laura Bozicevic, Thirumalai Ananthanpillai Supraja, Jonathan Hill, Prabha S. Chandra, Helen Sharp

**Affiliations:** ^1^Department of Biostatistics, Institute of Psychiatry, Psychology and Neuroscience, King’s College London, London, United Kingdom; ^2^Department of Primary Care and Mental Health, Institute of Population Health, University of Liverpool, Liverpool, United Kingdom; ^3^Department of Psychiatry, National Institute of Mental Health and Neurosciences, Bangalore, India; ^4^School of Psychology and Clinical Language Sciences, University of Reading, Reading, United Kingdom

**Keywords:** stroking, tactile stimulation, early maternal caregiving, gender, psychometric assessment, infant development

## Abstract

Differences in infant caregiving behavior between cultures have long been noted, although the quantified comparison of touch-based caregiving using uniform standardized methodology has been much more limited. The Parent-Infant Caregiving Touch scale (PICTS) was developed for this purpose and programming effects of early parental tactile stimulation (stroking) on infant hypothalamic-pituitary adrenal (HPA)-axis functioning (stress-response system), cardiovascular regulation and behavioral outcomes, similar to that reported in animals, have now been demonstrated. In order to inform future studies examining such programming effects in India, we first aimed to describe and examine, using parametric and non-parametric item-response methods, the item-response frequencies and characteristics of responses on the PICTS, and evidence for cross-cultural differential item functioning (DIF) in the United Kingdom (UK) and India. Second, in the context of a cultural favoring of male children in India, we also aimed to test the association between the sex of the infant and infant “stroking” in both cultural settings. The PICTS was administered at 8–12 weeks postpartum to mothers in two-cohort studies: The Wirral Child Health and Development Study, United Kingdom (*n* = 874) and the Bangalore Child Health and Development Study, India (*n* = 395). Mokken scale analysis, parametric item-response analysis, and structural equation modeling for categorical items were used. Items for two dimensions, one for stroking behavior and one for holding behavior, could be identified as meeting many of the criteria required for Mokken scales in the United Kingdom, only the stroking scale met these criteria in the sample from India. Thus, while a comparison between the two cultures was possible for the stroking construct, comparisons for the other non-verbal parenting constructs within PICTS were not. Analyses revealed higher rates of early stroking being reported for the United Kingdom than India, but no sex differences in rates in either country and no differential sex difference by culture. We conclude that PICTS items can be used reliably in both countries to conduct further research on the role of early tactile stimulation in shaping important child development outcomes.

## Introduction

Caregiving is an essential feature of mammalian development, not just for ensuring survival *via* the provision of infant nutrition, but for promotion of their physiological, cognitive, and social-emotional development. Caregiving broadly includes feeding, caring for physical health, providing sensory and intellectual stimulation, ensuring safety, providing emotional warmth and affection, comfort when distressed, and responding to the infants needs and communications in a timely fashion. Studies of early parental caretaking behavior in humans have typically focused on complex and often multidimensional observational indices of the quality of interaction between parent and infant during caregiving, like “maternal sensitivity” (Tryphonopoulos et al., 2014), or they require parents to report their beliefs about parenting practices and behaviors (Winstanley and Gattis, 2013). Alternatively, more limited specific domains of caretaking such as feeding (Hughes et al., 2005, 2012; Thompson et al., 2009), sleeping, or soothing (Morrell and Cortina-Borja, 2002) are the focus. Quantitative study of the role of touch as part of caregiving for the development of human infants has been much more limited, and cross-cultural comparison of these behaviors even rarer despite research suggesting that practices may differ considerably.

The work in this paper was conducted as part of a larger cross-cultural study designed to identify shared and distinctive risks and protective factors (including parenting behavior) for child mental health and cognitive development in the United Kingdom and India, comparing findings from two-cohort studies. A key first step was therefore to assess parenting behaviors in a standardized manner and formally test the psychometric characteristics of the scale used in both cultural settings [the Parent-Infant Caregiving Touch Scale (PICTS); Koukounari et al., 2015, this journal]. The main body of this paper addresses two aims. We first examine the measurement invariance of the PICTS between the United Kingdom and India. We next examine associations between responses on the PICTS and demographic characteristics of the child and parent, for subscales where measurement invariance was demonstrated across settings. In particular, in the light of cultural favoring of male compared to female children in India, we examine whether a difference in parental investment is identifiable in infancy.

We begin by summarizing previous research in animals and humans that provides evidence for the developmental importance of early touch-based caregiving. This research motivated the original development of the PICTS parent-report questionnaire. We next examine the evidence for potential cross-cultural differences in touch-based parenting and outline the rationale for a gender-based comparison in the current study.

In animals, work on rodents has long recognized the function of licking and grooming behavior by mothers to their pups in stimulating digestion. However, there is now overwhelming evidence for the broader importance of touch in rodents, arising from the detailed understanding of the epigenetic mechanism that links maternal licking and grooming behavior (Meaney and Szyf, 2005), or even just mechanical tactile stimulation with a brush (Imanaka et al., 2008), to hypothalamic-pituitary-adrenal (HPA) axis programming and its consequent long-term impact on anxiety-related behavior (Meaney and Szyf, 2005).

In humans, touch-based caregiving has largely focused on the effects of early skin-to-skin contact. In premature babies, early skin-to-skin contact is thought to be important because it has been found to promote optimal physiological outcomes (e.g., growth, autonomic functioning, and organized sleep) and to stimulate digestion (Bergman et al., 2004; Chhugani and Sarkar, 2014; Feldman et al., 2014; Chi Luong et al., 2016). It has also been shown to facilitate more responsive and synchronous mother-child interaction (Bigelow et al., 2010; Feldman et al., 2014; Vittner et al., 2018) and to support emotional development (e.g., efficient emotion regulation and reduced infant stress-response), and later cognitive abilities such as sustained attention and control (Feldman et al., 2002, 2014; Gonya et al., 2017). However, a recent Cochrane review of the use of skin-to-skin contact in healthy newborn infants supported its use to promote breastfeeding but concluded that the evidence for benefits to healthy infants in terms of greater stability of the cardio-respiratory system and higher blood glucose levels was based on only a few heterogeneous studies with small samples (Moore et al., 2016) making the clinical significance of the findings hard to determine at that time. However, a few studies have also examined the role of early tactile stimulation (indexed by parental stroking of the infant) in HPA-axis programming and find some parallels with the stress-response pattern seen in rodents (Sharp et al., 2012; Pickles et al., 2017) and comparability of the underlying epigenetics, including observed alterations in methylation of the glucocorticoid receptor gene (Murgatroyd et al., 2015). Specifically, early parental stroking moderated the impact of prenatal risk on early infant temperament and physiological response to social stress at 7 months of age and later emotional and behavioral development in the preschool period (Sharp et al., 2012; Pickles et al., 2017). There can therefore be little doubt that touch is an important sensory exposure that may shape both animal and human development.

Differences between cultures in caregiving behavior have long been noted, though their quantified comparison using uniform standardized methodology has been much more limited. A number of studies have examined early maternal sensitivity (i.e., contingent and appropriate responses to children’s cues) across cultures and there is some debate regarding the reliable application of the observation systems devised in western settings and their application to non-western settings (Keller et al., 2018; Mesman, 2018; Mesman et al., 2018). Keller et al. (2018) argue against the universality of maternal sensitivity and claim that the main components of sensitive responsiveness (i.e., the child taking the lead, the child’s point of view as primary, and the turn-taking structure of interactions) reflect a Westernized way of conceptualizing caregiving. Whereas, those who support the universality of maternal sensitivity assert that while its centrality and its manifestations can vary across cultures (i.e., caregivers might use different behaviors to respond to their children, such as vocal and tactile actions, repositioning of the infant, and following of child’s gaze), sensitive responses can be found in every culture (Mesman, 2018; Mesman et al., 2018). This work highlights the importance of considering the validity of measuring different aspects of caregiving within different cultural settings and of determining their consequences for infant development in that setting. It highlights the possibility that different caregiving behaviors may be observed which serve a similar developmental function across cultures, and vice versa. Furthermore, behaviors may be observed but at different frequencies reflecting differences in cultural expression, with differing long-term consequences for development.

Previous work has shown that caregiving arrangements in traditional societies often prioritize body contact (Richman et al., 1992), whereas face-to-face exchange and object play have been observed to be less pronounced (Greenfield and Suzuki, 1998). Cross-cultural observational studies have shown that when non-western mothers interact with their infants, they use more so called “proximal behaviors”: they might touch or stimulate the infant with hands (e.g., fondling or patting) or the face (e.g., kissing) or by body contact as opposed to “distal behaviors” (e.g., talking, looking, and smiling; Kärtner et al., 2008). In many non-western cultures, including India, cultural practices often include the provision of body stimulation in the form of a daily massage to the new born infant (Chaturvedi et al., 2020). Such practices are likely to have evolved within cultures and been passed through generations as they are believed to be associated with more favorable developmental outcomes for the infant. Traditional systems of medicine in India advocate the use of daily massage with oil as an integral part of infant care. Massage typically involves stroking of the infant’s legs, feet, back, abdomen, and head with oil. However, the few studies in India evaluating outcomes following touch-based interventions conducted to date have all been very small scale. The majority has reported increased weight gain in those infants massaged daily for a short period of weeks compared to controls. This has been observed in premature infants (Agarwal et al., 2000; Mathai et al., 2001; Arora et al., 2005; Sankaranarayanan et al., 2005) and healthy babies born at term (Sankaranarayanan et al., 2005), with one study reporting improved neurobehavioral outcomes also (Mathai et al., 2001). All these studies taught the massage technique to mothers to deliver as part of the study procedure and none assessed frequency of naturally occurring infant stroking between mother and infant.

The naturally occurring practice of early tactile stimulation suggests that touch is culturally valued and that a first step in understanding the impact of infants’ early exposure to touch-based caregiving would be to assess variations in exposure within the context of familial caregiving. To do this, one must be able to quantify variations in a standardized manner, such as frequency of occurrence, for different infants. First, in the context of their primary caregiver relationship, and then only later in relation to the broader caregiving system. In this paper, we describe the pattern of responses on the Parent-Infant Caregiving Touch Scale (Koukounari et al., 2015) in two cultures, the United Kingdom and India. While some aspects of early caregiving touch may occur at similar frequencies across these two different cultures, others may not. For instance, in India traditional, caregiving almost universally favors the deliberate use of early infant massage with oils in contrast to the United Kingdom where cultural beliefs do not emphasize the primary importance of this form of early touch. The study will yield important information about the natural occurrence of a range of touch-based parenting behaviors, including stroking, which may have particular importance for later infant outcomes.

As well as the study of cultural variations in caregiving touch behaviors, the study of gender-specific touch is of interest from a variety of perspectives. In South Asian populations, male children are strongly favored over females, and this preference may be relevant both to the mothers’ own childhood experiences of being parented and to their subsequent attitudes and behavior toward their own infant. Particularly in the age group 1–4 years, India has the most anomalous levels of excess female mortality in the world (Kashyap, 2019). However, in a context of formally declared gender-neutral policies and legislation, the reporting or display of gender-based differences in research may be suppressed on higher level self-report indices of caring, where norms of social desirability are common. The study of differences in touch behavior, that is arguably less subject to social-desirability norms, may expose such otherwise hidden gender differences. In the current study, we hypothesized that male infants would receive higher levels tactile stimulation compared to female infants in India, but that this would not hold true for the United Kingdom. Another reason for examining the role of gender in this study stems from our previous work on mothers and babies in the United Kingdom which suggests that, as in other mammals, touch-based programming of the HPA-axis may have gender-specific effects with stronger effects in females (Sharp et al., 2015). This study used the PICTS to assess parental stroking of the infant at 9 weeks of age.

In a previous paper, we reported on the factor structure and longitudinal invariance of the Parent-Infant Caregiving Touch Scale (PICTS) in the United Kingdom Wirral Child Health and Development Study (WCHADS) of women and their children, recruited in pregnancy (Koukounari et al., 2015). In this paper, we further examine the properties of this same instrument in WCHADS and the Bangalore/Bengaluru Child Health and Development Study (BCHADS), a parallel study of Indian mothers and infants. We describe and examine using parametric and non-parametric item-response methods, the item-response frequencies and characteristics, and evidence for cross- cultural differential item functioning (DIF). Examining DIF allows us to answer the question of whether reported differences in parental behaviors can be attributed to true differences in behavior rather than cultural differences in the psychometric properties of the items. Finally, we describe and test the association with a number of characteristics of mothers and children, examining in particular differences between male and female infants in the United Kingdom and India in the receipt of stroking, the touching behavior we believe to be most relevant to HPA-axis programming.

## Materials and Methods

### Participants

The BCHAD study in India is the first longitudinal investigation in India with a core focus on mental health in the mother and the child from pregnancy onward. The Indian cohort was recruited in three Primary Health Care centers delivering maternity care in Bangalore (Banashankari, Siddhaiah road hospital and N.R. Colony hospital). All pregnant women aged 18 years and above in the first or second trimester of pregnancy, were approached over a 20-month period. 84 women were excluded from postnatal follow-up as the pregnancy was high risk or the baby did not survive. A total of 825 women remained eligible for postnatal follow-up. Of these 825 women, 395 mothers completed the PICTS scale at 8 weeks of age and represent the sample for the current report. In pregnancy, 83.7% mothers were homemakers, only a quarter had completed secondary level of education or higher, and the average family income was Rs. 10,000 (~£105 per month). This is comparable to similar urban settings in India (Indian Census Bureau, 2011).

The Wirral Child Health And Development Study (WCHADS) is a United Kingdom cohort comprising of first-time mothers aged 18 years and above and their partners, who were approached and recruited at a local ante natal clinic, the sole public provider on the Wirral peninsula, during 2007 and 2008. A total of 1,233 women had a live singleton baby and were eligible for postnatal follow-up. From these 1233 women, 874 mothers completed the PICTS scale at 9 weeks of age (during 2015 and 2016) and represent the sample for the current report (see Koukounari et al., 2015 for more details). Although generally typical of the United Kingdom population, the Wirral population under-represents the ethnic diversity of the United Kingdom as a whole.

Baseline sociodemographic data for the cohort participants included in the analyses of this paper are reported in Table 1.

**Table 1:** Demographic characteristics for those with a PICTS assessment in United Kingdom and India.

	India (BCHADS)	UK (WCHADS)
Maternal Age mean (SD) [n]	23.0 years (3.36) [393]	28.2 years (5.69) [873]
Child’s Age mean (SD) [n]	10.8 weeks (3.7) [393]	9.0 weeks (2.8) [872]
Female % [n]	48.1% [395]	50.6% [874]
First born % (r/n)	43.3% [393]	100.0% [874]
Marital Status [n]	[393]	[857]
Married %	100.0%	47.6%
Cohabiting %	0.0%	34.4%
Single/Other %	0.0%	18.0%
EPDS total score (SD) [n]	1.49 (3.92) [391]	5.82 (4.64) [856]
Religion Hindu v Muslim % [n]	85.0% [390]	NA
Family Type Nuclear v Joint/Extended % [n]	44.0% [392]	NA
Education % [n]		
above secondary - India (age 13 or 14) [n]	27.5% [393]	NA
above secondary – United Kingdom (age 18) [n]	NA	64.9% [857]

### Ethical Approvals and Consent Procedure

The United Kingdom study was approved by the Cheshire North and West Research Ethics Committee (UK) on the 27th June 2006. The Indian study was approved by the National Institute for Mental Health and Neuroscience (NIMHANS Ethics Committee on the 2nd of July 2015) and the University of Liverpool Ethics Committee (1st March 2016). All women gave written informed consent to take part.

### Measures

The Parent-Infant Caregiving Touch scale (PICTS) is a 12-item parent-report scale designed to assess common caregiving behavior for parents of young infants. Four items assess tactile stimulation in the form of stroking. These ask how often the mother strokes her baby’s back, head, tummy, arms, and legs. Remaining items reflect various other forms of touch or communication. Mothers responded to all items on a 5-point scale ranging from 0 to 4 with labels: never, rarely, sometimes, often, and a lot. Using factor analysis on the United Kingdom sample used here, Koukounari et al. (2015) showed the psychometric structure of the 12 items mapped on to three domains of maternal behavior (four items each): stroking (stroke back, head, tummy, arms, and legs), holding (hold, cuddle, pick up, and rock), and affective communication (kissed, talked to, watched, and left baby to lie down), which showed adequate model fit. Internal reliability was good with the polychoric ordinal alpha over 0.87 and 0.89 for infants at 5 and 9 weeks, respectively.

The scale was administered at around 8–12 weeks of age in the Indian cohort and data from the 9–12 weeks assessment in the United Kingdom sample was used. In the United Kingdom sample, women completed the scale as a self-report postal questionnaire, whereas in India, due to low levels of literacy, the scale was administered orally by a researcher in a face-to-face visit at the home. In both countries, women completed this scale as part of a larger set of study measures at this time point. The PICTS scale items and the abbreviation used to refer to each item in this paper are given in Table 2.

**Table 2:** The Parent-Infant Caregiving Touch Scale (PICTS).

How often do you find yourself doing each of the following things with your baby? *(Please circle one response)*						Abbreviation for item
	Never	Rarely	Sometimes	Often	A lot	
I hold my baby	1	2	3	4	5	hold
I pick my baby up	1	2	3	4	5	pick up
I talk to my baby	1	2	3	4	5	talk
I cuddle my baby	1	2	3	4	5	cuddle
I rock my baby	1	2	3	4	5	rock
I kiss my baby	1	2	3	4	5	kiss
I stroke my baby’s tummy	1	2	3	4	5	tummy
I stroke my baby’s back	1	2	3	4	5	back
I stroke my baby’s face.	1	2	3	4	5	face
I stroke my baby’s arms or legs	1	2	3	4	5	limbs
I watch my baby	1	2	3	4	5	watch
I leave her/him to lie down, (e.g., in pram/ cot/basket/mat)	1	2	3	4	5	lie

The Edinburgh Postnatal Depression Scale (EPDS) is a 10-item self-report scale designed to assess perinatal depressive symptomatology (Cox et al., 1987) and it was administered contemporaneously with PICTS. Mothers answered each item indicating how they have felt during the previous week on a set of four answers that were subsequently coded by researchers on a scale between 0 and 3. Each item on the EPDS has a different response set with severity of depression reflected in a higher score. For example, the statement, “I have felt sad or miserable” appears with a response set “Yes, most of the time = 3,” “Yes, quite often = 2,” “Not very often = 1,” and “No, not at all = 0.” The higher the total score, the more depressive symptomatology women experienced. This scale was included to enable us to examine whether responses on the PICTS varied as a function of contemporaneous maternal mental health or sociodemographic factors.

### Statistical Analysis

We divided the analysis into three steps. In the first two steps, we compared the measurement properties of PICTS in the two cohorts before, in the third step, modeling differences in the caregiving behavior of the two cohorts. We used Mokken Scale Analysis (Mokken, 1971; Stochl et al., 2012), a form of non-parametric item response theory (IRT),” to investigate the formal measurement properties and structure of the PICTS in the United Kingdom and Indian cohorts. This included the use of kernel smoothed IRT (Mazza et al., 2012) and automated item selection procedure (AISP). The advantage of non-parametric over parametric IRT is that it gives an easily interpretable assessment of item covariance while making few assumptions about the data structure (Meijer and Baneke, 2004). Since we wanted to compare caring behavior across cohorts and gender (within cohorts), we then assessed differential item functioning (DIF). Here, we apply the MIIO test (Manifest Invariant Item Ordering, Ligtvoet et al., 2010) and report the H^T^ coefficient that evaluates the accuracy by which the respondents order the set of items (Ligtvoet et al., 2010). Although the monotone homogeneity (MH) model is sufficient for assessing the psychometric structure of the PICT scale, IIO means the order of items is the same across all values of latent trait, which would argue against DIF. We assessed the reliability of the dimensions using Mokken Scale Rho (Sijtsma and Molenaar, 1987). We applied parametric IRT models to test for gender differences as this provided formal methods to statistically test DIF. Finally, we applied latent regression to determine whether caregiving behavior differed across gender, whether associations with gender differed across country and conducted similar tests for the demographic variables and concurrent postnatal depression in Table 1.

## Results

**Figure 1:**
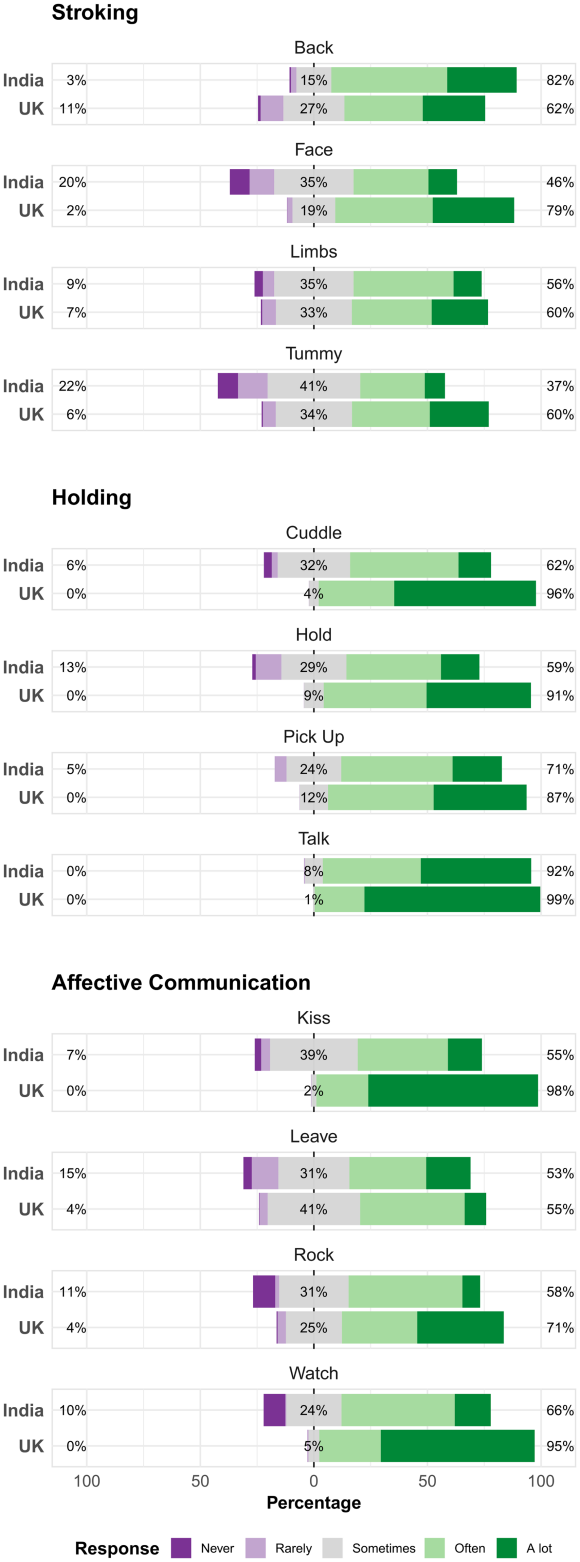
Distribution of responses to PICTS items according to country cohort. The distributions are centered at the middle category “Sometimes.” Percentages are given for the center response, high response (greater than sometimes), and low response (less frequently than sometimes).

Table 1 gives descriptive statistics for the two cohorts. Figure 1 shows each item of the PICTS questionnaire and response distributions for both the United Kingdom and Indian cohorts. For all items apart from the item relating to stroking the infant’s back, WCHADS mothers self-reported a higher frequency of the behavior than those from BCHADS.

### Mokken Scale Analysis

Reported in Table 3, in the United Kingdom sample, two of the three dimensions previously identified by confirmatory factor analysis (Koukounari et al., 2015) showed strong Mokken scalability, with *H* = 0.59 for the stroking and 0.67 for the holding dimensions (generally a minimum value of 0.3 for H is needed to confirm items as a scale: 0.3 to <0.4 indicate a weak scale, 0.4 to <0.5 a moderate scale, and 0.5 and greater a strong scale; Stochl et al., 2012). The 4-item affective communication dimension fell below the 0.3 criterion. However, it formed a weak to moderate scale (*H* = 0.38) if the “leave” item was removed. Using the automated item selection procedure—AISP (Mokken, 1971) on all 12 items (equivalent to exploratory factor analysis but identifying subsets of items forming Mokken scales rather than factors), identified 2 strong scales, the first consisting of the stroking items and a second of the holding items with the addition of “kiss” (*cf.* Table 2). For the rest of this analysis of the United Kingdom cohort, we only considered these two subscales.

**Table 3:** Mean response, scalability coefficients (Loevinger’s H), and Dimension Loadings (D) for weak (H > 0.3) and strong (H > 0.5) scale requirements for the United Kingdom (one and two dimension solutions) and Indian cohorts (three and four dimension solutions) as identified by Mokken automated item scale procedure (AISP).

		UK					India								
				AISP Dimensions “Loadings”						AISP Dimensions “Loadings”					
				H > 0.3	H > 0.5					H > 0.3				H > 0.5	
	Item	Mean	Item Scalability	D1	D1	D2	Mean	Item Scalability	D1	D2	D3	D4	D1	D2	D3
D1: Stroking	Tummy	3.80	0.61	0.48		0.61	3.15	0.65	0.6				0.65		
	Back	3.76	0.54	0.43		0.54	4.08	0.56	0.54				0.56		
	Face	4.13	0.60	0.45		0.60	3.30	0.70	0.65				0.70		
	Limbs	3.78	0.62	0.47		0.62	3.56	0.68	0.62				0.68		
	Overall		0.59					0.65							
D2: Holding	Hold	4.37	0.72	0.46	0.7		3.61	0.30				0.56		0.56	
	Cuddle	4.28	0.69	0.46	0.69		3.87	0.24				0.56		0.56	
	Pick up	4.77	0.72	0.41	0.53		4.40	0.26	0.38						
	Rock	4.58	0.58	0.51	0.7		3.67	0.13		0.33					
	Overall		0.67					0.23							
D3: Affective Communication	Kiss	4.04	0.30	0.45	0.55		3.44	−0.12			0.67				0.67
	Talk	4.72	0.34	0.41			3.60	0.08		0.33					
	Watch	4.63	0.26	0.32			3.62	−0.06			0.67				0.67
	Leave	3.61	0.13				3.54	0.06							
	Overall		0.25					−0.02							

There were no statistically significant deviations from monotonicity for these two subscales. Further, the tests of local independence showed no violations. Neither the stroking nor holding dimensions showed significant violations of the invariant item ordering test. However, the H^T^ statistic, for which larger values indicate less likelihood of DIF, showed little support for invariant item ordering for the stroking scale (*H*_T_ = 0.17) but more support for the holding scale (*H*^T^ = 0.46). Reliability was high for both dimensions (Mokken Scale Rho for stroking = 0.83 and holding = 0.85).

In BCHADS, the same analyses indicated strong scalability for the stroking dimension (*H* = 0.65), strong scale local independence, no violations of monotonicity, and strong support for IIO (*H*^T^ = 0.63). However, the holding and affective communication dimensions showed only a very weak scalability (holding *H* = 0.23, affective communication H was slightly negative). Applying AISP identified multiple dimensions at the different lower bounds of H. When requiring H > 0.3 for all scales, four dimensions were selected with one 5-item dimension (the four stroking items with the addition of “pick up”) and three 2-item scales. Requiring H > 0.5, three dimensions were identified; one four item scale (stroking), two items from the holding dimension (hold and cuddle), and two items from affective communication dimension (kiss and watch). None of the other items formed a Mokken scale. For the two item scales, the affective communication dimension showed no violations of monotonicity nor local independence. The holding dimension, while passing the local independence test, showed a significant violation of monotonicity, where the probability of rating the “hold” item in the highest categories decreased with increasing score for the rest of the items (whereas they should increase together).

In summary, the Mokken scale analysis indicated the scale has a different structure across cohorts with only the stroking dimension identified in both cohorts. The holding and affective communication items did not form respective scales for the India cohort (the “leave” item did not scale in either of the cohorts). Given this non-compatibility of scale structure, for the cross-cultural comparison, we focus on the stroking dimension alone.

### Differential Item Functioning of the Stroking Scale by Country

Comparing scalability coefficients between cohorts, items were roughly similar with large overlap between 95% CI. The exception was the face item (*cf.* Table 3) which showed no overlap. There was stronger scaling for the face item in India (i.e., fewer Guttman errors) than in the UK. The proportion of responses per category tended to differ between the two cohorts but were similar for the “limbs” item for all but the probability of responding with category 5 (A lot).

**Figure 2:**
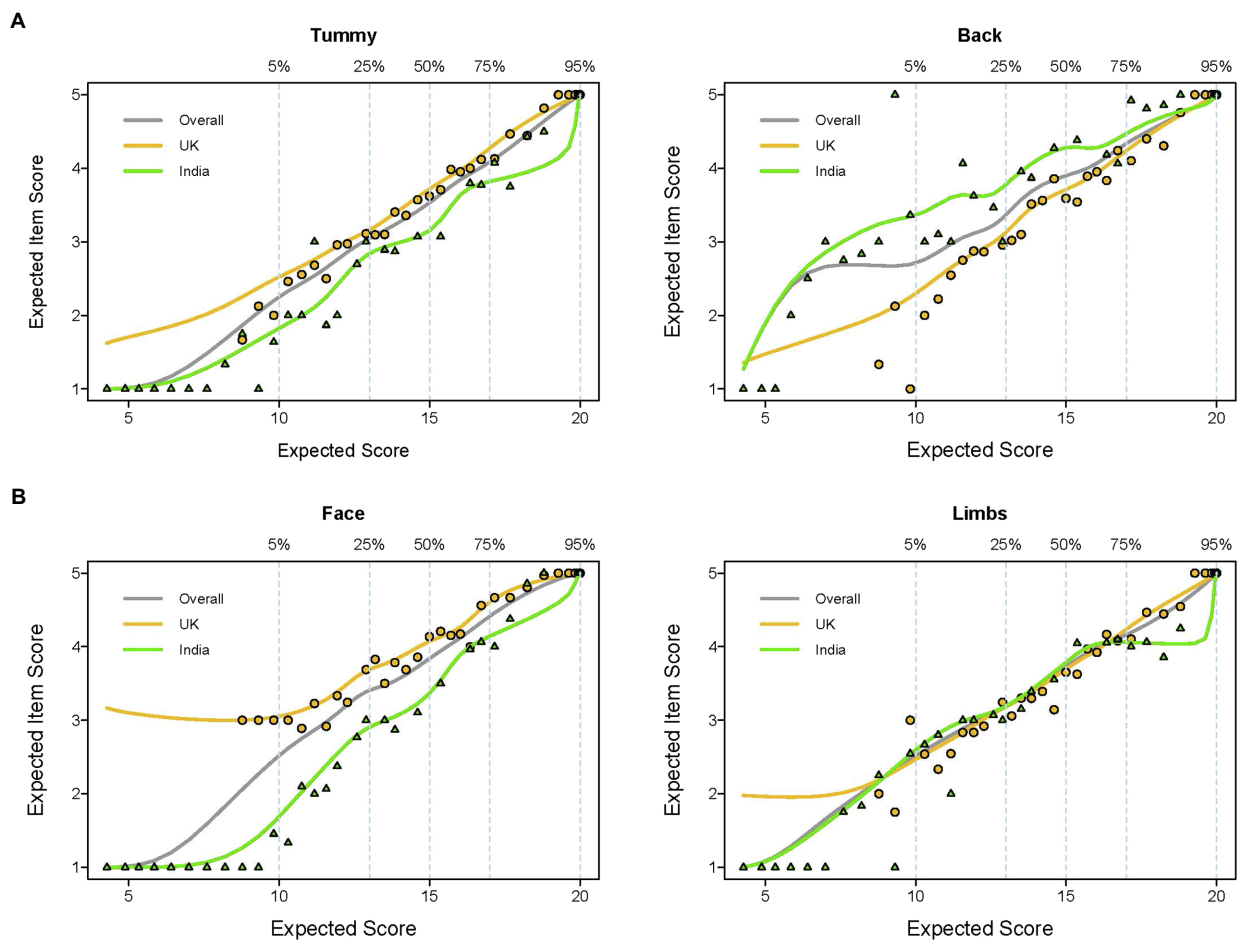
Comparison of Item-Response Functions (IRF) for the four stroking items for India versus United Kingdom mothers. Expected item scores are presented as a function of expected total scores illustrating two issues: **(A)** monotonicity (the likelihood of items being endorsed with increases in the latent propensity of stroking behavior) and **(B)** whether the latent propensity to stroke shows the same pattern of item response in the different countries. The percentage scale indicates the cumulative proportion of the sample that have the corresponding total scores and the points represent groups of participants (grouped by ordinal ability). The IRFs are compared in each country, with Indian IRFs in green and the United Kingdom in amber.

Figure 2 compares the expected item scores as a function of expected total score. Respondents in India reported less back stroking than mothers in the United Kingdom and the absence of face stroking among low strokers in India is evident. The “limbs” item performed the most similar across countries and was thus used as the anchor item for the parametric DIF analysis below.

### Parametric Analysis of Differential Item Functioning

Parametric graded membership models were used for further comparison. In the first model, the limbs item was fixed between country with all other items and structural parameters allowed to differ between groups. Although RMSEA was acceptable at 0.034, model fit was not particularly good (*G*^2^ = 1188.9, *p* < 0.001). Fixing the slopes and intercepts of the remaining three items across groups worsened fit in all three cases (Tummy: *χ*^2^ = 69.9, *p* < 0.001, Back: *χ*^2^ = 111.4, *p* < 0.001, Face: *χ*^2^ = 179.3, *p* < 0.001). Item DIF was present for all three items. To investigate further, we next tested model fit for the slope of items. While there was no difference for tummy (*χ*^2^ = 1.8, *p* = 0.357) or back (*χ*^2^ = 1.4, *p* = 0.357) there was for face (*χ*^2^ = 46.4, *p* < 0.001). In the next step, the face item was allowed to vary between groups and the intercepts for the remaining two items were sequentially fixed. Constraining the intercepts of both items to be the same resulted in poorer model fit (Tummy: *χ*^2^ = 72.1, *p* < 0.001, Back: *χ*^2^ = 112.7, *p* < 0.001).

Having established that only partial metric invariance could be assumed, we tested the structural parameters for invariance. Constraining the factor variance to be 1 in both groups made no difference to model fit (*χ*^2^ = 0.03, *p* = 0.860). Respondents in India showed no difference in variability in stroking behavior relative to the United Kingdom, but the factor mean was different between groups (*χ*^2^ = 95.7 *p* < 0.001) with the Indian cohort reporting lower stroking behavior than the United Kingdom (*β* = −0.66; 95% CI, −0.79, −0.52).

Finally, we examined differential test functioning using the partial metric invariance model. Though the expected score for the back item was greater for India than the United Kingdom (*p* = 0.027), the difference was relatively small, equating to an overall points difference of 0.14. Table 4 shows the comparison of scalability coefficients and partial metric IRT model parameters for the United Kingdom and India.

**Table 4:** India-UK comparison between non-parametric (Mokken scaling) and parametric (IRT) DIF for the stroking dimension including: non-parametric scalability coefficients H_i_ (and 95% CI), cumulative probability of responses (*Cum* Pr), and parametric estimates with 95% CI of slope (a1) and intercept coefficients (d1–d4) for the IRT Graded Response Model (GRM).

Variables		Mokken						Parametric IRT				
Item	Group	H_i_	Cum Pr_1_	Cum Pr_2_	Cum Pr_3_	Cum Pr_4_	Cum Pr_5_	a1	d1	d2	d3	d4^*^
Back	India	0.47 (0.39, 0.55)	1.00	0.99	0.89	0.62	0.27	1.89 (1.66, 2.12)	6.99 (5.72, 8.25)	5.15 (4.48, 5.82)	2.59 (2.23, 2.96)	−0.58 (−0.89, −0.26)
	United Kingdom	0.47 (0.43, 0.52)	1.00	0.99	0.97	0.82	0.31	1.89 (1.66, 2.12)	5.87 (5.14, 6.59)	3.02 (2.71, 3.33)	0.860 (0.650, 1.08)	−1.46 (−1.70, −1.22)
Face	India	0.63 (0.57, 0.68)	1.00	1.00	0.98	0.79	0.36	8.99 (2.45, 15.5)	8.94 (2.85, 15.0)	2.13 (0.120, 4.14)	−5.78 (−9.42, −2.14)	
	United Kingdom	0.52 (0.47, 0.56)	1.00	0.91	0.80	0.46	0.13	2.44 (2.07, 2.81)	5.76 (5.07, 6.44)	2.47 (2.12, 2.82)	−0.92 (−1.19, −0.66)	
Limbs	India	0.63 (0.57, 0.69)	1.00	1.00	0.94	0.60	0.24	3.00 (2.64, 3.35)	7.37 (6.60, 8.13)	5.02 (4.52, 5.52)	1.18 (0.890, 1.47)	−2.39 (−2.74, −2.05)
	United Kingdom	0.6 (0.56, 0.63)	1.00	0.96	0.91	0.56	0.12	3.00 (2.64, 3.35)	7.37 (6.60, 8.13)	5.02 (4.52, 5.52)	1.18 (0.890, 1.47)	−2.39 (−2.74, −2.05)
Tummy	India	0.62 (0.56, 0.68)	1.00	1.00	0.94	0.60	0.26	2.96 (2.60, 3.32)	5.00 (4.37, 5.63)	3.22 (2.73, 3.71)	0.13 (−0.29, 0.54)	−3.14 (−3.73, −2.55)
	United Kingdom	0.59 (0.55, 0.63)	1.00	0.91	0.78	0.37	0.09	2.96 (2.60, 3.32)	8.27 (7.08, 9.46)	5.03 (4.49, 5.58)	1.12 (0.82, 1.42)	−2.08 (−2.42, −1.73)

* The d4 intercept on the face item could not be estimated as only 1 participant in the United Kingdom responded Never to stroking the face. For this item, the never and not often categories were merged.

### Differential Item Functioning by Gender

For the UK, the scalability coefficients showed little difference for males and female infants, indicating similar discrimination of stroking items by gender (*cf.* Table 5).

**Table 5:** Female-Male comparison of stroking scale items for the United Kingdom cohort, by non-parametric (Mokken scaling) and parametric (IRT) DIF including: non-parametric scalability coefficients H_i_ (and 95% CI), cumulative probability of responses (*Cum* Pr), and parametric estimates with 95% CI of slope (a1) and intercept coefficients (d1–d4) for the IRT Graded Response Model (GRM).

Variables		Mokken						Parametric IRT				
Item	Group	H_i_	Cum Pr_1_	Cum Pr_2_	Cum Pr_3_	Cum Pr_4_	Cum Pr_5_	a1	d1	d2	d3	d4^*^
Back	Female	0.53 (0.46, 0.6)	1.00	0.98	0.88	0.60	0.26	1.97 (1.59, 2.35)	5.49 (4.59, 6.39)	2.96 (2.49, 3.42)	0.730 (0.420, 1.05)	−1.65 (−2.01, −1.28)
	Male	0.56 (0.48, 0.63)	1.00	1.00	0.90	0.64	0.29	2.07 (1.58, 2.57)	6.90 (5.38, 8.42)	3.26 (2.72, 3.80)	1.07 (0.670, 1.46)	−1.30 (−1.70, −0.89)
Face	Female	0.58 (0.52, 0.65)	1.00	1.00	0.97	0.80	0.35	2.31 (1.82, 2.80)	5.37 (4.50, 6.24)	2.41 (1.94, 2.88)	−0.960 (−1.32, −0.60)	
	Male	0.62 (0.56, 0.69)	1.00	1.00	0.98	0.79	0.37	2.73 (2.04, 3.42)	6.38 (5.20, 7.56)	2.58 (1.99, 3.18)	−0.860 (−1.33, −0.38)	
Limbs	Female	0.59 (0.53, 0.65)	1.00	0.99	0.93	0.61	0.25	3.18 (2.59, 3.76)	8.73 (7.32, 10.2)	5.21 (4.46, 5.95)	1.13 (0.710, 1.56)	−2.35 (−2.84, −1.86)
	Male	0.64 (0.59, 0.7)	1.00	1.00	0.94	0.59	0.24	3.18 (2.59, 3.76)	8.73 (7.32, 10.2)	5.21 (4.46, 5.95)	1.13 (0.710, 1.56)	−2.35 (−2.84, −1.86)
Tummy	Female	0.59 (0.53, 0.65)	1.00	1.00	0.93	0.60	0.24	2.65 (2.11, 3.19)	7.87 (6.16, 9.57)	4.57 (3.82, 5.32)	0.99 (0.60, 1.39)	−2.12 (−2.60, −1.64)
	Male	0.63 (0.57, 0.69)	1.00	1.00	0.94	0.60	0.28	2.97 (2.21, 3.74)	8.20 (6.41, 9.99)	5.11 (4.17, 6.05)	1.15 (0.620, 1.67)	−1.78 (−2.35, −1.22)

* *The d4 intercept on the face item could not be estimated as only 1 participant in the* United Kingdom *responded Never to stroking the face. For this item, the never and not often categories were merged*.

For the parametric IRT analysis, we again used the limbs item as the anchor. Model fit for the United Kingdom sample was acceptable in terms of RMSEA (*p* = 0.03). There was no DIF for the remaining items (all p’s > 0.05) nor overall difference at the test level (*p* = 0.424). With items held constant between groups, there was no difference in factor means (*χ*^2^ = 0.53 *p* = 0.468) nor variance between males and females (*χ*^2^ = 0.08 *p* = 0.782).

For the India cohort (c.f. Table 6), there was also little gender difference in scalability coefficients and kernel smoothed IRF’s (Table 5). The parametric IRT analysis showed acceptable model fit for a between group model with limb as the anchor item, according to the RMSEA criterion (*p* = 0.03). No gender DIF was apparent for the remaining items (all p’s > 0.05) nor at test level (*p* = 0.099). With cross-group item parameters fixed, there was no difference in factor means (*χ*^2^ = 0.23, *p* = 0.633) and nor variance (*χ*^2^ = 1.34, *p* = 0.237).

**Table 6:** Female-Male comparison of stroking scale items for the India cohort, by non-parametric (Mokken scaling) and parametric (IRT) DIF including: non-parametric scalability coefficients H_i_ (and 95% CI), cumulative probability of responses (*Cum* Pr), and parametric estimates of with 95% CI slope (a1) and intercept coefficients (d1–d4) for the IRT Graded Response Model (GRM).

Variables		Mokken						Parametric IRT				
Item	Group	H_i_	CumPr(1)	CumPr(2)	CumPr(3)	CumPr(4)	CumPr(5)	a1	d1	d2	d3	d4^*^
Back	Female	0.53 (0.4, 0.66)	1.00	1.00	0.97	0.83	0.31	1.42 (1.02, 1.82)	6.44 (4.37, 8.52)	4.43 (3.49, 5.38)	1.99 (1.55, 2.43)	−1.06 (−1.43, −0.70)
	Male	0.58 (0.48, 0.69)	1.00	0.99	0.97	0.81	0.30	1.72 (1.16, 2.27)	5.87 (4.29, 7.46)	4.26 (3.35, 5.18)	1.80 (1.30, 2.31)	−1.30 (−1.76, −0.83)
Face	Female	0.72 (0.64, 0.79)	1.00	0.91	0.81	0.45	0.15	32.9 (−29.7, 95.5)	26.4 (−23.5, 76.4)	−2.94 (−11.1, 5.25)	−32.6 (−95.5, 30.4)	
	Male	0.69 (0.62, 0.77)	1.00	0.92	0.80	0.46	0.11	4.98 (2.74, 7.21)	3.53 (2.03, 5.02)	−0.58 (−1.59, 0.42)	−6.19 (−8.48, −3.91)	
Limbs	Female	0.69 (0.59, 0.78)	1.00	0.96	0.90	0.56	0.12	2.62 (2.11, 3.13)	5.49 (4.61, 6.37)	3.93 (3.29, 4.58)	0.33 (−0.07, 0.73)	−3.54 (−4.13, −2.95)
	Male	0.67 (0.58, 0.76)	1.00	0.97	0.92	0.57	0.13	2.62 (2.11, 3.13)	5.49 (4.61, 6.37)	3.93 (3.29, 4.58)	0.33 (−0.07, 0.73)	−3.54 (−4.13, −2.95)
Tummy	Female	0.62 (0.53, 0.72)	1.00	0.89	0.78	0.40	0.10	2.61 (2.05, 3.18)	3.86 (3.10, 4.61)	2.35 (1.79, 2.90)	−0.54 (−0.97, −0.12)	−3.84 (−4.64, −3.05)
	Male	0.67 (0.59, 0.75)	1.00	0.93	0.78	0.34	0.08	3.52 (2.39, 4.66)	4.92 (3.68, 6.16)	2.50 (1.64, 3.36)	−1.44 (−2.24, −0.65)	−5.39 (−6.77, −4.01)

* *The d4 intercept on the face item could not be estimated as only 1 participant in the* United Kingdom *responded Never to stroking the face. For this item, the never and not often categories were merged.*

### Comparing Stroking Behavior Between Country and Gender

**Figure 3:**
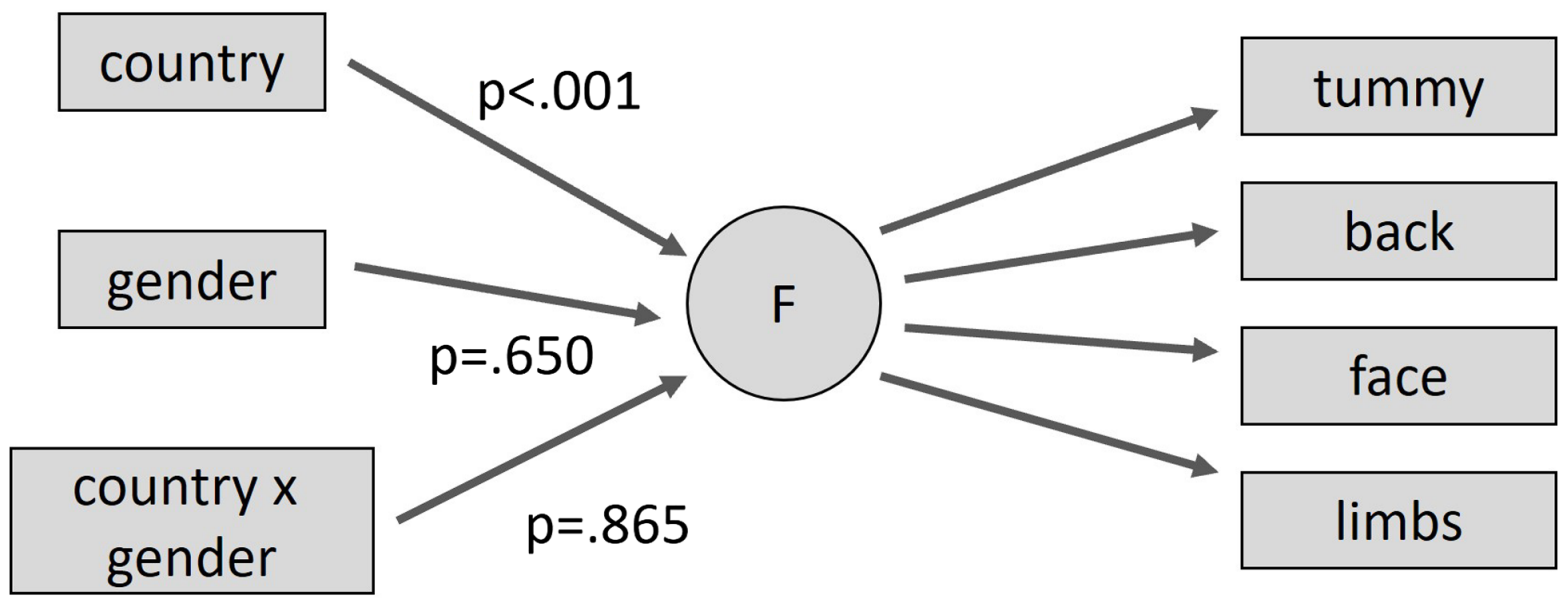
Testing structural differences in maternal stroking behavior by country and gender (males and United Kingdom taken as reference category for dummy variables).

As we achieved at least partial metric invariance by country, a latent regression model of the form of Figure 3 was fitted jointly to both cohorts and the interaction between country and gender for stroking behavior was examined. For the United Kingdom, stroking frequency was slightly higher for males than females but this difference was not significant, *β* = 0.045, *p* = 0.554. More importantly, although this difference was incrementally reduced for the Indian cohort relative to the United Kingdom (−0.09; 95%CI: −0.34, 0.16), the interaction between gender and country was not significant. The effects are summarized in Figure 3. As the interaction was not significant, we refitted the model with simple effects of country and gender. Not surprisingly, stroking behavior was reported as significantly less frequent in India than the United Kingdom (difference estimate −0.66; 95%CI: −0.8, −0.52, *p* < 0.001).

### Associations With Demographic Characteristics

Finally, we extended the model of Figure 3 to examine the association of various other characteristics with the level of stroking. Stroking increased with the age of the infant (*p* = 0.002), but this appeared to be the similar across countries (*p* = 0.383) and had no effect on the absence of gender effects. There was no association with maternal age (*p* = 0.351) nor differences by country. For variables that were not comparable across countries or unique to one country, there was an effect for higher maternal education being associated with more stroking of marginal significance in India (*p* = 0.055) but significantly less stroking in the United Kingdom (*p* = 0.007). There was no difference by religion (India Muslim/Hindu *p* = 0.630), parity (*p* = 0.370), nor family type (nuclear v joint/extended *p* = 0.620) in India, and no difference by marital status in the United Kingdom (married v cohabiting v single/other 2df *p* = 0.227). There was no association with contemporaneous depression as measured by the EPDS in India (*p* = 0.093) nor the United Kingdom (*p* = 0.956).

## Discussion

Before apparent differences in reported behavior across cultures can be attributed to real differences in the levels of underlying constructs, we must first show that the measuring instrument being used performs equivalently in those cultures. While this may be obvious, in comparisons with parenting it is also rarely highlighted as requiring serious methodological investigation (e.g., Lansford, 2021). It is all too easy to assume not only that the familiar constructs from the considerable literature on parenting in developed western societies are universal, but that these constructs are both understood and made evident in behavior in the same way. This study examined a measure of largely non-verbal parental caregiving, the PICTS, and found that while items for two dimensions, one for stroking behavior and one for holding behavior, could be identified as meeting many of the criteria required for Mokken scales in the United Kingdom, only the stroking scale met these criteria in the sample from India. Thus, while a comparison between the two cultures is possible for the stroking construct, comparisons for the other non-verbal parenting constructs was not possible. Similar measurement invariance concerns also need to be addressed before comparing the parenting of male and female children within each culture. Here, we found no evidence of differences in the ways in which any of the constructs were measured for male and female infants.

Much of the literature on parenting in western societies has focused on meeting the supposed needs of the child for healthy physical, emotional, cognitive, and social development. In practice, the parenting of children must also fit into the other social roles that are expected of parents (Costigan et al., 2003). The constraints that these can impose are perhaps no more evident than in the simple proximity that is allowed and considered acceptable, from the 90% of time spent in skin-to-skin contact of some hunter-gatherers (Diamond, 2012) to its unacceptability even for feeding in many western work and public spaces (Adesoye et al., 2017). Although differences in understanding of the needs of infants may contribute, it is perhaps as much in the detailed cultural variations in social roles of mothers and how parenting can be fitted within the demands of these other roles that explains how the specific behaviors of the PICTS questionnaire items do not coalesce into constructs common across cultures. The exception is the stroking construct which perhaps stands out as being, at least superficially, inessential. We might speculate that instead, stroking is carried out spontaneously and naturally as a method of soothing or conveying affection across cultures, or as part of a more deliberate infant-focused ritual when the demands from other roles are set aside.

Focusing on the stroking scale for which direct cross-cultural comparison seemed justified, the final analyses examined the different levels of the scales between cultures, genders, and culture-by-gender; this last being potentially indicative of increased parental investment in boy infants compared to girls that might be expected in India. Our findings were very clear, with higher rates of early stroking being reported for the United Kingdom than India, but no sex differences in rates in either country and no differential gender difference by culture. This is consistent with recent findings of a decline in intra-household sex discrimination found for the under-5 mortality of opposite-sex twins in southern India (Kashyap and Behrman, 2020).

The findings suggest that an examination of the role of stroking behaviors in early maternal caregiving and their developmental consequences in India is likely to be methodologically sound using the PICTS measure. Previous research by our group has reported moderation of prenatal stress effects by tactile stimulation (assessed using the PICTS) in the first few weeks of life, on infant negative emotionality and heart rate variability to a social stressor at 7 months of age and on later emotional and behavioral development in the preschool period (Sharp et al., 2012; Pickles et al., 2017) in the United Kingdom and to underlying methylation of the glucocorticoid receptor gene (Murgatroyd et al., 2015). That this measure of physical parenting, while showing clear individual differences, and varying with the infant’s age, does not appear to be influenced by maternal age, social or religious background, or contemporaneous depressive symptoms of the mother, strengthens the case for these findings not being due to confounder bias. The findings within this methodological paper enable us to confidently move on to examine whether similar protective effects of stroking in early life are evident in India in the same way as the United Kingdom. This work on the performance of the PICTS scale can also be used as a model to guide the future approach to assessment of non-verbal caregiving practices in other cultural settings.

## Strengths and Limitations

The studies, while large for this field of research, may not be sufficient to provide definitive answers, and particularly in relation to the absence of sex differences, require replication. The analytic approach used in this two-cohort study is thorough and exposed multiple differences in the measurement properties of the PICTS between the two countries. These differences cannot be unequivocally attributed to differences in parenting and response culture, since the mode of assessment, text presentation in the United Kingdom vs. verbal presentation in India, may account for some differences with face-to-face responding arguably being more vulnerable to response bias. It is also possible that, though both cohorts were general populations recruited in pregnancy, differences in sampling frames (first born in the United Kingdom, all births in India) and differential attrition may have contributed to some differences. However, these weaknesses in design are likely of more concern for comparison of means by country, than for differences by sex within countries. Thus, our conclusion concerning no difference between the United Kingdom and India in sex-based differences is likely robust.

## Future Work

We are mindful of the fact that future work should extend the examination of naturally occurring stroking behaviors by primary caregivers to include that reported by other key caregivers in order to represent the overall exposure that infants receive. For instance, in India, shared caregiving especially for infants is the cultural norm with many key figures such as grandmothers or aunties contributing significantly to early infant care. This may potentially explain why the comparison of frequency of stroking between United Kingdom and India showed lower levels of stroking behavior overall from mothers in India. In order to characterize the levels of tactile stimulation received by Indian infants, one should ideally gather data from other primary caregivers in addition to mothers in a shared-caregiving context.

Finally, to further characterize the level of tactile stimulation received by infants in United Kingdom and India, future studies should record whether or not the infant has received early skin-to-skin contact or infant massage as part of an early intervention or caregiving practice locally. We are aware that baby massage groups have become a relatively common feature of perinatal support within the United Kingdom (Asmussen and Brim, 2018) and it is also common practice in India for daily massage with oils to be given for the first 40 days of life by grandmothers or local paid birth attendants (Dai; Chaturvedi et al., 2020). A recent large-scale study in two states (Maharashtra and Madhya Pradesh) in India reported infant massage practice to be the norm rather than the exception with 93.8% of mothers reporting its practice. It should be noted, however, that in line with the findings of our study, mothers reported that the practice was equivalent for female and male babies (Chaturvedi et al., 2020). Although in our Indian sample frequency of stroking did not differ by infant sex or parity of the mother, future research should investigate whether a more complex relationship between caregiving practices and infant sex may exist. Gender-based caregiving may be dependent on a third contextual factor, the number of existing male and female children in the family.

## Conclusion

Before cross-cultural differences in parenting and developmental processes can be claimed, evidence should first be presented that the assessment tools perform in the same way in both cultures. We have examined the role of touch in the parenting of infants in two culturally distinct settings using PICTS, a parent-report questionnaire developed for use in the United Kingdom. We showed that the instrument did not have the same psychometric properties in the two countries, with only some subscales being replicated and only partial invariance within replicated subscales. The infant stroking subscale, previously identified as important for HPA-axis programming in the United Kingdom sample, showed sufficient invariance to allow a valid cross-cultural comparison. While rates of stroking were lower in India than the United Kingdom, there was no evidence of the expected higher rates of stroking of male infants in the Indian cohort with no sex differences being evident in either country. We are yet to examine whether the role of stroking in HPA-axis programming is common across the two cultures. More rigorous evidence is also required before we can recommend the use of clinical interventions, such as the promotion of baby massage, that might play a meaningful role in child development. Future research also needs to better substantiate the measurement equivalence of other aspects of parenting, both touch-based and more generally, to compliment the assessment of stroking behavior.

## Data Availability Statement

The datasets presented in this article are not readily available because of ethical constraints. Supporting data are available to *bona fide* researchers on approval of an application for access. Further information about the data and conditions for access are available at the University of Liverpool Research Data Catalogue: https://doi.org/10.17638/datacat.liverpool.ac.uk/564. Requests to access the datasets should be directed to HS, hmsharp@liverpool.ac.uk.

## Ethics Statement

The studies involving human participants were reviewed and approved by the Cheshire North and West Research Ethics Committee (UK) on the 27th June 2006 and by the National Institute for Mental Health and Neuroscience (NIMHANS Ethics Committee on the 2nd of July 2015) and the University of Liverpool Ethics Committee (1st March 2016). The patients/participants provided their written informed consent to participate in this study.

## Author Contributions

JHo, AP, HS, LB, PC, and TS wrote the paper. JHo and AP conducted the data analysis. HS, PC, JHi, AP, and TS designed and conducted the study. All authors contributed to the article and approved the submitted version.

## Funding

The WCHADS study was funded by the UK Medical Research Council (grant G0400577 to JHi, HS, and AP) and the BCHADS study was funded jointly by UK Medical Research Council and Indian Council for Medical Research (grants MR/N000870/1 and ICMR/MRC-UK/2/M/2015-NCD-1 to HS, PC, JHi, and AP). AP and JHo were partially funded by NIHR Biomedical Research Centre at South London and Maudsley NHS Foundation Trust and King’s College London. AP is partially supported by NIHR NF-SI-0617-10120. The views expressed are those of the authors and not necessarily those of the funders.

## Conflict of Interest

The authors declare that the research was conducted in the absence of any commercial or financial relationships that could be construed as a potential conflict of interest.

## Publisher’s Note

All claims expressed in this article are solely those of the authors and do not necessarily represent those of their affiliated organizations, or those of the publisher, the editors and the reviewers. Any product that may be evaluated in this article, or claim that may be made by its manufacturer, is not guaranteed or endorsed by the publisher.
